# Cardiopulmonary Function Assessment in Children With Pulmonary Valve Stenosis

**DOI:** 10.3389/fped.2021.802645

**Published:** 2022-01-14

**Authors:** Li Yun Teng, Sen Wei Tsai, Chun Yuan Hsiao, Wei Hung Sung, Ko Long Lin

**Affiliations:** ^1^Department of Physical Medicine and Rehabilitation, Taichung Tzu Chi Hospital, Taichung, Taiwan; ^2^Department of Orthopedic Surgery, Chiayi Chang-Geng Memorial Hospital, Chiayi, Taiwan; ^3^Ministry of Health and Welfare, Department of Rehabilitation Medicine, Taoyuan General Hospital, Taoyuan, Taiwan; ^4^Department of Physical Medicine and Rehabilitation, Kaohsiung Veteran General Hospital, Kaohsiung, Taiwan; ^5^School of Medicine, College of Medicine, Kaohsiung Medical University, Kaohsiung, Taiwan; ^6^School of Medicine, College of Medicine, National Yang Ming Chiao Tung University, Hsinchu, Taiwan

**Keywords:** cardiopulmonary exercise testing, pulmonary valve stenosis, exercise capacity, pulmonary function test, transthoracic echocardiogram

## Abstract

**Objective:**

Pulmonary valve (PV) stenosis affects cardiac pulmonary function and exercise performance. A cardiopulmonary exercise test (CPET) combined with a transthoracic echocardiogram (TTE) can measure exercise performance, disease progression, and treatment effects. We assessed the exercise capacity in children with PV stenosis by conducting CPET and TTE.

**Methods:**

From 2005 to 2021, 84 patients with PV stenosis aged 6–18 years were enrolled; 43 were treated with balloon pulmonary valvuloplasty (BPV) (Group A), and 41 received follow-up care (Group B), and their CPET and pulmonary function test results were compared with 84 healthy, matched individuals (Control). We also conducted TTE to compare the peak pulmonary artery pulse wave velocity and pulmonary valve (PV) area before and after catheterization and follow-up care.

**Results:**

There were no significant differences among the CPET parameters of the patient groups and controls in anaerobic metabolic equivalent (MET) (group A: 6.44 ± 0.58; group B: 6.28 ± 0.47, control: 6.92 ± 0.39, *p* = 0.110), peak MET (group A: 9.32 ± 0.74; group B: 9.13 ± 0.63; control: 9.80 ± 0.52, *p* = 0.263), and heart rate recovery (group A: 28.04 ± 4.70; group B: 26.44 ± 3.43, control:26.10 ± 2.42, *p* = 0.718). No significant differences were found in the pulmonary functions between the three groups. The pulmonary artery pulse wave velocity significantly decreased after catheterization (3.97 ± 1.50 vs. 1.95 ± 0.94, *p* < 0.0001), but not after follow-up care (1.67 ± 0.77 vs. 1.75 ± 0.66, *p* = 0.129). The pulmonary vale area significantly improved in group A (0.89 ± 0.71 vs. 1.16 ± 0.58, *p* < 0.0001), whereas only insignificant progression of PV stenosis was observed in group B (1.60 ± 0.64 vs. 1.57 ± 0.65, *p* = 0.110).

**Conclusions:**

Patients treated with BPV had a similar exercise capacity with that of patients under follow-up care and the healthy controls. Larger or multi-center studies should be conducted to confirm the physical fitness of pediatric patients with PV stenosis after management.

## Introduction

Pulmonary valve (PV) stenosis is a heart valve defect resulting in right ventricular outflow obstruction at the pulmonic valve (1, 2). It occurs in 1 per 2,000 live births and accounts for 8% of congenital heart diseases. The severity of PV stenosis is determined by Doppler echocardiography with a peak gradient across obstruction. A transvalvular pressure gradient <36 mm Hg (peak velocity <3 m/s) indicates mild PV stenosis, while 36–64 mm Hg (peak velocity 3–4 m/s) and >64 mm Hg (peak velocity >4 m/s) are moderate and severe stenosis, respectively (3). Disease management is based on severity; patients with mild PV stenosis usually have a benign natural evolution without progression (4, 5), receive echocardiographic follow-up care, and do not require further intervention. However, severe PV stenosis may progress during childhood (5), and interventions, such as catheter intervention or surgical valvotomy, are recommended. Indications for catheterization included PV stenosis patients with valves which are not dysplastic (BPV, balloon pulmonary valvuloplasty) and with peripheral PV stenosis (balloon angioplasty). As for surgical indication, they recommended surgical valvotomy for patients with subinfundibular or infundibular PV stenosis and hypoplastic pulmonary annulus, with dysplastic pulmonary valves, and for patients with associated severe pulmonary regurgitation or tricuspid regurgitation (3). The long-term prognosis of patients receiving proper management is excellent, with high survival rates and rare complications (6–8). However, existing studies measure outcomes based on hemodynamic change rather than functionally assessing physical capacity after the procedure.

Cardiopulmonary exercise testing (CPET) is considered the gold standard for assessing exercise capacity. CPET provides non-invasive, dynamic measurements that can be used for diagnostic, prognostic, and evaluative purposes (9–11). Current studies lack data regarding the exercise capacity of PV stenosis patients who underwent BPV. Measuring the maximum oxygen consumption (VO_2_ max) during a progressive cardiopulmonary exercise test is widely accepted for assessing aerobic fitness (12).

In this study, we evaluated the functional outcome of patients who underwent BPV to determine if PV stenosis patients receiving proper management could have similar exercise capacity with that of healthy individuals. Further, we aimed to provide more evidence regarding the influence of PV stenosis on exercise capacity in children.

## Materials and Methods

### Patient Selection and Data Collection

We retrospectively reviewed the medical records of children with isolated PV stenosis obtained from the pediatric outpatient department of Kaohsiung Veteran General Hospital in Taiwan. Ninety nine patients with PV stenosis were enrolled between January 2005 and May 2021. Patients under 18 years who understood the treadmill exercise testing steps and completed the treadmill exercise testing without abnormal electrocardiographic findings or symptoms were included. Patients with other congenital heart diseases (e.g., ventricular septal defect, atrial septal defect, patent ductus arteriosus), current or history of arrhythmia, or missing data were excluded. All patients had classical form of pulmonary valve stenosis. The study subjects were divided into patients treated with BPV (Group A) and patients who only received regular echocardiographic follow-up care (Group B). Patients who underwent BPV were those with severe PV stenosis or moderate PV stenosis with progressive symptoms, where as those under follow-up care were considered mild or asymptomatic moderate PV stenosis. Our subjects were managed based on the current guideline (3). All of the patients in group A did not receive re-interventions. All patients underwent body composition measurement, followed by cardiopulmonary exercise testing and lung function tests.

To compare the CPET parameters of the patient population, a control population was selected from a database of healthy children aged under 18 years who underwent cardiopulmonary exercise testing at the Veteran General Hospital of Kaohsiung, in whom no cardiac anomalies or underlying diseases were diagnosed. The control population was selected by 1:1 matching of age, sex, and body mass index using MedCalc (version 14.12.0; MedCalc, Ostend, Belgium). Informed consent was obtained parents of all patients before the examinations.

### Treadmill Exercise Testing

Treadmill exercise testing is possible from the age of three according to the American College of Sports Medicine (ACSM) (13). All participants underwent symptom-limited cardiopulmonary exercise testing consisting of a treadmill, a flow module, a gas analyzer, and an electrocardiographic monitor (Metamax 3 B; Cortex Biophysik GmbH Co., Liepzig, Germany); we adopted the Bruce protocol. We monitored the oximeter, blood pressure, and heart rate during testing. The test was terminated when the patient developed subjectively unbearable symptoms, could no longer continue, or reached their maximum exercise, as indicated by ACSM (14). Oxygen consumption (VO_2_) was measured using the breath-by-breath method. Metabolic equivalent (MET) values [i.e., 3.5 mL of oxygen per kilogram of body mass per minute (15)] were calculated after measuring the VO_2_. The peak and anaerobic threshold (AT) MET were defined as the maximum MET and the MET at AT throughout the entire exercise test, respectively. The AT was determined by the ventilatory equivalents for oxygen ratio [i.e., the expired volume (VE)/VO_2_] and the ventilation/carbon dioxide production slope [i.e., VE/the volume of exhaled carbon dioxide (VCO_2_)] (16). The respiratory gas exchange ratio (RER) was calculated as VCO_2_/VO_2_. Heart rate recovery (HRR) was calculated as the maximum heart rate (HR) during the test minus the HR at 1 min after testing. The VO_2_ max was reached when the RER was >1.1, the peak HR was >200 bpm, and the HR was >85% of the age-predicted maximum (13, 17, 18).

### Pulmonary Function Testing

Pulmonary function tests were performed by spirometry at rest and included the forced vital capacity (FVC), the forced expiratory volume in 1 second (FVE1), and the maximal voluntary ventilation (MVV).

### Transthoracic Echocardiography

The peak pulmonary arterial pulse wave velocity, pulmonary valve area, and ejection fraction were measured using a standard transthoracic echocardiographic examination. All examinations were performed by pediatric cardiologists of the Veteran General Hospital of Kaohsiung using a sector probe with a more than 5-MHz frequency based on the standard measurement methods for pediatric echocardiograms outlined by the American Society of Echocardiography (19). The highest velocities and PV area obtained were included in the analysis and were compared pre- and post-management. Ejection fraction of at baseline was compared between group A and B.

### Statistical Analyses

Continuous data are presented as means ± standard deviations, and categorical variables are expressed as absolute numbers or percentages. The analysis of variance test with *post hoc* analysis by Student-Newman-Keuls test was used to compare differences between the study and control populations in the continuous variables of demographic and exercise parameters, while a Chi-square test was used to compare the categorical variables. A paired *t*-test was used to analyze the difference in peak pulmonary arterial pulse wave velocity and PV area obtained from transthoracic echocardiogram before and after PV stenosis management (i.e., BPV or follow-up care). An independent *t*-test was employed to compare ejection fraction in groups A and B at baseline. Statistical significance was set at *p* < 0.05. Analyses were performed using MedCalc (version 14.12.0; MedCalc, Ostend, Belgium).

## Results

### Patient Characteristics

**Figure 1:**
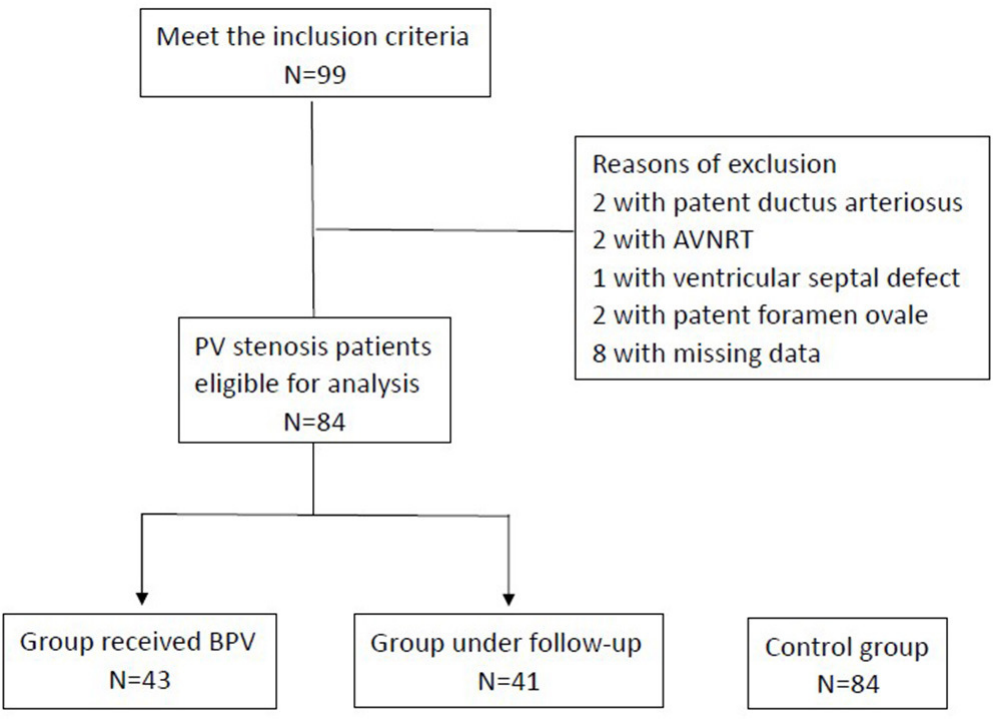
Process of patient selection.

In total, 99 patients met the inclusion criteria; two had patent ductus arteriosus, two had atrioventricular nodal reentry tachycardia, one had a ventricular septal defect, two had patent foramen ovale, and eight had missing data and were excluded. Figure 1 presents the patient selection process. Of the remaining 84 patients, 43 underwent BPV (Group A), and 41 underwent regular follow-up (Group B). The average age was 11.91 ± 3.82 years in Group A (42% were girls) and 11.95 ± 3.32 in group B (59% were girls). The baseline characteristics did not differ between the A, B, and Control groups (Table 1).

**Table 1:** Demographic characteristics.

	**Group A**	**Group B**	**Control group**	***P*-value**
Gender (male, %)	58%	41%	50%	0.462
Age (years)	11.91 ± 3.82	11.95 ± 3.32	11.95 ± 3.37	0.997
Height (cm)	147.76 ± 6.34	146.10 ± 5.53	148.78 ± 3.57	0.732
Body weight (Kg)	46.55 ± 6.23	44.97 ± 5.25	45.79 ± 3.37	0.914
BMI (Kg/m^2^)	20.44 ± 1.47	20.27 ± 1.35	20.19 ± 0.94	0.955
Body fat (%)	19.58 ± 3.18	22.46 ± 2.44	19.83 ± 1.89	0.232
Resting SBP (mmHg)	110.56 ± 4.03	112.73 ± 5.62	109.49 ± 2.49	0.466
Resting DBP (mmHg)	64.16 ± 2.93	64.28 ± 3.20	65.13 ± 1.80	0.807
Resting HR (bpm)	89.35 ± 4.92	85.56 ± 4.12	88.21 ± 3.05	0.459

*BMI, body mass index; DBP, diastolic blood pressure; HR, heart rate; SBP, systolic blood pressure. Group A, children with pulmonary stenosis receiving BPV; Group B, children with pulmonary stenosis simply receiving follow-up care*.

### Cardiopulmonary Exercise and Pulmonary Function Testing

Cardiopulmonary exercise testing was conducted for an average of 7.7 years in Group A (after BPV) and 9.2 years in Group B (follow-up care). All subjects who underwent cardiopulmonary exercise testing achieved a maximum level indicated by the RER (Group A: 1.14 ± 0.04; Group B: 1.18 ± 0.04; Control: 1.21 ± 0.02).

**Figure 2:**
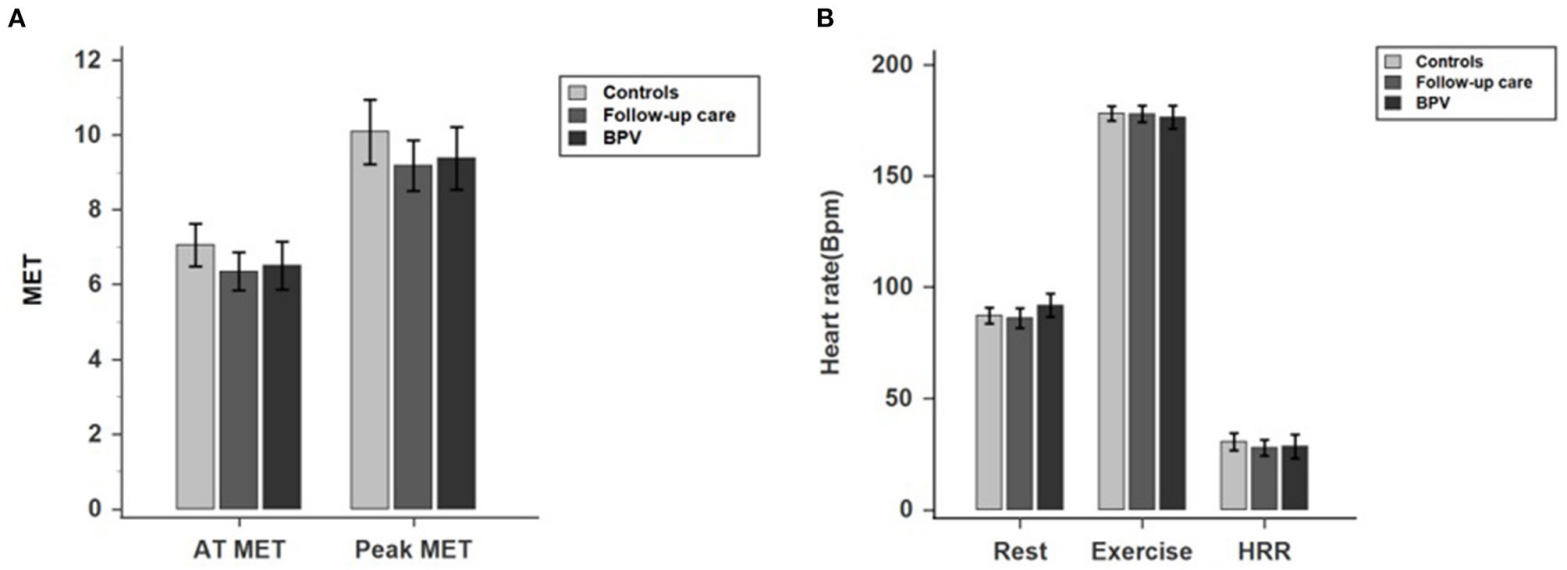
**(A,B)** Comparison of exercise parameters between patients receiving BPV, under follow-up care, and control group.

Patients receiving BPV had comparable exercise capacity with those in the follow-up care and control groups, as the AT MET (Group A: 6.44 ± 0.58; Group B: 6.28 ± 0.47; Control: 6.92 ± 0.39, *p* = 0.110), peak MET (Group A: 9.32 ± 0.74; Group B: 9.13 ± 0.63; Control: 9.80 ± 0.52; *p* = 0.263), peak VO_2_ (group A: 32.63 ± 8.38; Group B: 31.95 ± 7.12; Control: 34.30 ± 8.47; *p* = 0.263) and HRR (Group A: 28.04 ± 4.70; Group B: 26.44 ± 3.43; Control: 26.10 ± 3.42; *p* = 0.718) did not differ (Figure 2). Furthermore, the mile per hour (group A: 3.09 ± 1.48; Group B: 2.83 ± 1.45; Control: 3.32 ± 1.21; *p* = 0.201) and grade (group A: 13.26 ± 3.92; Group B: 12.91 ± 2.67; Control: 14.31 ± 3.26; *p* = 0.074) at peak exercise, peak HR (Group A: 174.88 ± 5.01; Group B: 177.78 ± 3.43; Control: 178.67 ± 1.66; *p* = 0.196), resting HR (Group A: 89.35 ± 4.92; Group B: 85.56 ± 4.12; Control: 88.21 ± 3.05, *p* = 0.459), peak systolic blood pressure (Group A: 164.02 ± 11.03; Group B: 163.51 ± 8.58; Control: 164.86 ± 7.15; *p* = 0.974), and peak diastolic blood pressure (group A: 84.42 ± 7.63; Group B: 85.12 ± 6.64; Control: 83.88 ± 4.27; *p* = 0.954). On the other hand, the pulmonary function did not differ between the groups. The results of cardiopulmonary exercise testing and pulmonary function and testing were listed in Table 2. Figure 2 presents the comparison of exercise parameters between patients receiving BPV, under follow-up care, and control group.

**Table 2:** Comparison of cardiopulmonary testing and pulmonary function testing.

	**Group A**	**Group B**	**Control group**	***P*-value**
AT MET	6.44 ± 0.58	6.28 ± 0.47	6.92 ± 0.39	0.110
AT HR, bpm	138.84 ± 4.50	141.12 ± 3.43	143.35 ± 2.32	0.124
Peak VE, L	41.10 ± 4.70	40.83 ± 4.32	44.09 ± 3.03	0.366
Peak MET	9.32 ± 0.74	9.13 ± 0.63	9.80 ± 0.52	0.263
Peak HR, bpm	174.88 ± 5.01	177.78 ± 3.43	178.67 ± 1.66	0.196
Peak RER	1.14 ± 0.04	1.18 ± 0.04	1.21 ± 0.02	0.001^*^
Peak SBP, mmHg	164.02 ± 11.03	163.51 ± 8.58	164.86 ± 7.15	0.974
Peak DBP, mmHg	84.42 ± 7.63	85.12 ± 6.64	83.88 ± 4.27	0.954
HRR at 1 min, bpm	28.04 ± 4.70	26.44 ± 3.43	26.10 ± 2.42	0.718
Peak VO_2_, ml/kg/min	32.63 ± 8.38	31.95 ± 7.12	34.30 ± 8.47	0.263
mph at peak exercise	3.09 ± 1.48	2.83 ± 1.45	3.32 ± 1.21	0.201
Grade at peak exercise, %	13.26 ± 3.92	12.91 ± 2.67	14.31 ± 3.26	0.074
FVC, L	2.56 ± 0.39	2.39 ± 0.28	2.39 ± 0.17	0.484
FVE1, L	2.43 ± 0.34	2.36 ± 0.59	2.23 ± 0.16	0.638
FEV1/FVC, %	86.58 ± 3.96	88.71 ± 2.59	90.47 ± 1.76	0.095
MVV, L	55.08 ± 7.79	50.91 ± 6.70	60.00 ± 5.16	0.123

*AT, anaerobic threshold; AT MET, metabolic equivalent at the point of anaerobic threshold; DBP, diastolic blood pressure; HR, heart rate; HRR, heart rate recovery; Peak MET, maximal metabolic equivalent during exercise testing; RER, respiratory exchange threshold; SBP, systolic blood pressure; mph, mile per hour; FVC, forced vital capacity; FVE1, forced expiratory volume in 1 s; MVV, maximal voluntary ventilation*.

* *p-value < 0.05. Group A, children with pulmonary stenosis receiving BPV; Group B, children with pulmonary stenosis simply receiving follow-up care*.

### Transthoracic Echocardiography

Transthoracic echocardiography was conducted at the same period as cardiopulmonary exercise testing. Patients with moderate PV stenosis, as identified by peak PV velocity between 3 and 4 m/s, accounted for 57% in group A and 27% in group B. Patients who underwent BPV had more severe disease at the baseline, but the pulmonary arterial pulse wave velocity significantly decreased after the procedure (before BPV: 3.97 ± 1.50; after BPV: 1.95 ± 0.94; *p* < 0.0001). The peak pulmonary arterial pulse wave velocity before and after follow-up care did not differ (before follow-up care: 1.59 ± 0.79; after follow-up care: 1.75 ± 0.72; *p* = 0.642). The pulmonary valve area significantly improved in group A (0.89 ± 0.71 vs. 1.16 ± 0.58, *p* < 0.0001), whereas only insignificant progression of PV stenosis was observed in group B (1.60 ± 0.64 vs. 1.57 ± 0.65, *p* = 0.110). Ejection fraction (EF) at baseline was similar between group A and B(Group A: 72.41 ± 11.72; Group B: 69.17 ± 11.90; *p* = 0.932). Table 3 presents the results of transthoracic echocardiography.

**Table 3:** Comparison of pulmonary arterial pulse wave velocity before and after management.

	**Pre-treatment**	**Post-treatment**	***P*-value**
**PAV(m/sec)**			
Group A	3.97 ± 1.50	1.95 ± 0.94	<0.0001^*^
Group B	1.67 ± 0.77	1.75 ± 0.66	0.129
**PV area (cm** ^**2**^ **)**			
Group A	0.89 ± 0.71	1.16 ± 0.58	<0.0001^*^
Group B	1.60 ± 0.64	1.57 ± 0.65	0.110
	Group A	Group B	*p*-value
EF (%)	72.41 ± 11.72	69.17 ± 11.90	0.932

*PAV, peak pulmonary arterial pulse wave velocity; PV, pulmonary valve; EF, ejection fraction*.

* *p-value < 0.05. Group A, children with pulmonary stenosis receiving BPV; Group B, children with pulmonary stenosis simply receiving follow-up care*.

## Discussion

This retrospective cohort study found that the VO_2_ max, peak and AT HR, and HRR did not differ between the experimental and control groups. The peak pulmonary artery pulse wave velocity of patients who underwent BPV significantly decreased after the procedure, but only a mild, statistically insignificant pulmonary artery pulse wave velocity increase was observed for those who underwent follow-up care. On the other hand, the PV area greatly improved after BPV, but insignificantly decreased after follow-up care.

Our study showed that pediatric patients with PV stenosis who underwent BPV could have comparable exercise capacity with that of those under follow-up care and healthy individuals. Patients before BPV had more severe PV stenosis, as indicated by greater preoperative peak pulmonary arterial pulse wave velocity and smaller PV area, which was addressed after the procedure. For patients under follow-up care, disease progression was benign, with no considerable increase in peak pulmonary arterial pulse wave velocity and change in PV area. Therefore, we can be more confident as to the good outcome of PV stenosis patients undergoing proper management.

Several reports have shown that patients with PV stenosis have a reduced exercise tolerance. For example, Goldberg et al. (20) studied the maximum exercise capacity in children with PV stenosis, reporting that children with pulmonary stenosis had a lower relative maximum endurance time than children without cardiac anomalies. Ikkos et al. (21) attempted to correlate working capacity with the PV area, and patients with a PV area index <0.3 cm^2^ /m^2^ had a lower maximum working capacity. They concluded that the diminished exercise tolerance in children with PV stenosis was related to reduced stroke volume and cardiac output. However, existing data discussing exercise tolerance in these patient groups mainly included unoperated PV stenosis.

Studies on the exercise capacity of pediatric patients treated with BPV are limited. In 1984, Rocchini et al. (22) attempted to compare the exercise hemodynamics of patients undergoing BPV and performed a study in which two nine-year-old children exercised on a supine bicycle ergometer immediately before and immediately after BPV. They found a significant decrease in the maximal right ventricular systolic pressure and the right ventricle to pulmonary artery peak pressure gradient. However, their study was limited by a small sample size and lacked standardized protocols for cardiopulmonary testing at that time.

Our findings were consistent with previous data regarding the excellent outcomes of BPV. Voet et al. (23) evaluated the long-term prognosis of treated PV stenosis by reviewing patients treated surgically and with BPV; the patients treated with BPV were followed up for a median of 6 years. They found that both surgery and BPV were safe and successful in relieving the transpulmonary gradient. However, this previous study has focused on hemodynamic changes rather than functional assessments of physical capacity. Our research directly measured exercise capacity with a comprehensive cardiopulmonary assessment, providing further evidence that indicated that patients should undergo catheterization-based treatment.

The cardiopulmonary testing in our patient group showed a similar capacity to that of their healthy peers. Current suggestions regarding the physical activities of patients with PV stenosis by Graham et al. (24) recommended that asymptomatic patients with a systolic Doppler gradient below 40 mmHg and normal RV function should be encouraged to engage in normal activity, including sport. However, in patients with a stenosis gradient above 40 mmHg, competitive sports should be discouraged or (re)intervention should be considered beforehand. In our study, the cardiopulmonary testing in our patient group showed a similar physical capacity to that of their healthy peers. The exercise parameters implied that children with PV stenosis might be able to engage in physical education classes at school after proper management and cardiac follow-up. Furthermore, previous data indicated that reasons for reduced physical activities were multifactorial and might be related to restrictions from worried parents (25, 26). Our findings provide more convincing data, which might lessen the concerns of parents that may preclude physical activities or recreational sports for their children.

This study has several limitations. First, we adopted test termination criteria according to ACSM guidelines (13). However, to our knowledge, no studies have discussed the criteria for achieving VO_2_ max during exercise testing in children with congenital heart disease. Second, surgical valvotomy is an option for managing severe PV stenosis, especially for those with dysplastic pulmonary valves, hypoplastic annulus, or main pulmonary atresia, or for those with subvalvular or supravalvular PV stenosis (6, 27). However, patients who underwent surgical valvotomy were not included in our study. Furthermore, most patients in the follow-up care group were those with mild PV stenosis. A selection bias of low-risk patients should be considered when interpreting the study results. Lastly, this was a single-center study with a relatively small sample size. The results might not apply to the entire nation. Larger nationwide or multi-center studies among Taiwanese patients are required.

## Conclusions

Patients with PV stenosis receiving catheterization could have comparable exercise capacity with that of those under follow-up care and even healthy controls. Despite these encouraging results, future studies should encompass larger patient groups to further clarify the exercise capacity of children with PV stenosis.

## Data Availability Statement

The original contributions presented in the study are included in the article/supplementary material, further inquiries can be directed to the corresponding author.

## Ethics Statement

The studies involving human participants were reviewed and approved by Institutional Review Board of Kaohsiung Veteran General Hospital (VGHKS 17-CT11-11). Written informed consent to participate in this study was provided by the participants' legal guardian/next of kin.

## Author Contributions

LT, KL, and ST contributed to conception and design of the study. CH organized the database. WS performed the statistical analysis. LT wrote the first draft of the manuscript. All authors contributed to the article and approved the submitted version.

## Conflict of Interest

The authors declare that the research was conducted in the absence of any commercial or financial relationships that could be construed as a potential conflict of interest.

## Publisher's Note

All claims expressed in this article are solely those of the authors and do not necessarily represent those of their affiliated organizations, or those of the publisher, the editors and the reviewers. Any product that may be evaluated in this article, or claim that may be made by its manufacturer, is not guaranteed or endorsed by the publisher.
